# Supplementary material for: The Therapeutic Potential of Naturally Occurring Peptides in Counteracting SH-SY5Y Cells Injury
Source: Int J Mol Sci. 2022 Oct 4;23(19):11778. doi: 10.3390/ijms231911778 (PMC9569762; doi:10.3390/ijms231911778)
Supplement: Supplementary file 1 [file ijms-23-11778-s001.zip › ijms-1910258-supplementary.pdf]

International Journal of
Molecular Sciences

MDPI

Supplementary Materials

The Therapeutic Potential of Naturally Occurring Peptides in Counteracting SH-SY5Y Cells Injury

Renata Perlikowska [1,*], Joana Silva [2], Celso Alves [2], Patrícia Susano [2] and Rui Pedrosa [3]

1 Department of Biomolecular Chemistry, Faculty of Medicine, Medical University of Lodz, 92-215 Lodz, Poland
2 MARE—Marine and Environmental Sciences Centre, Politécnico de Leiria, 2520-630 Peniche, Portugal
3 MARE—Marine and Environmental Sciences Centre, ESTM, Politécnico de Leiria, 2520-641 Peniche, Portugal
* Correspondence: renata.perlikowska@umed.lodz.pl

Contents
- Antioxidant activity of the peptides (0.1-10 μM) evaluated through the DPPH method (Figure S1).
- Antioxidant activity of peptides and standards (Table S1).

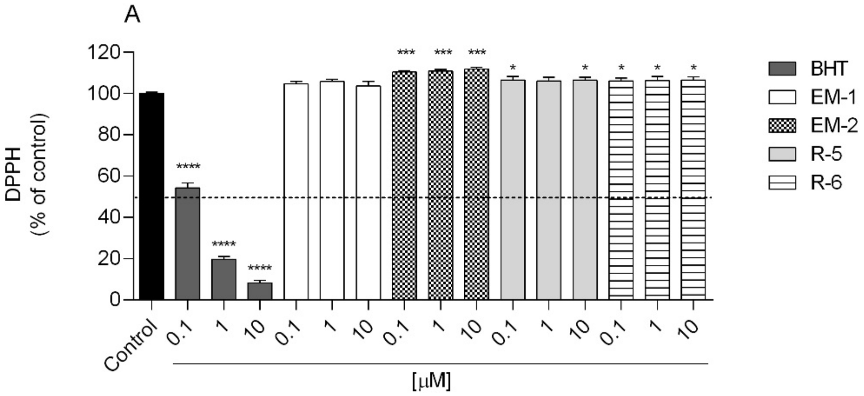

Figure S1. Antioxidant activity of the peptides (0.1-10 μM) evaluated through the DPPH methods. BHT (butylated hydroxytoluene) used as a standard. The values in each column represent the mean ± standard error of the mean (SEM) of 3 independent experiments. Symbols represent significant differences (ANOVA, Dunnett's test) *p < 0.05 and **** < 0.0001 when compared to control.

Table S1. Antioxidant activity of peptides and standards.

Peptides	DPPH[a,*]	FRAP[b]	ORAC[c]
**EM-1**	> 10	0.80 ± 0.18	9115 ± 112.9
**EM-2**	> 10	0.44 ± 0.15	2692 ± 197.8
**R-5**	> 10	0.64 ± 0.18	2720 ± 129.7
**R-6**	> 10	1.10 ± 0.20	2789 ± 131.6
**BHT**	0.13 (0.09 – 0.19)	-	136.4 ± 9.09

The values in table represent the mean ± standard error of the mean (SEM) from 3 independent experiments. [a] radical scavenging activity (EC_{50} μM); [b]μmol TE/g extract; [c]μM $FeSO4$/g extract. *EC_{50} values (μg/mL) were determined for the samples that scavenged DPPH radical over 50%.
